# Low-temperature RWGS enhancement of Pt_1−*n*_Au_*n*_/CeO_2_ catalysts and their electronic state

**DOI:** 10.1039/d3ra06635e

**Published:** 2023-10-06

**Authors:** Taigo Onodera, Tatsuya Miyake, Masatoshi Sugimasa

**Affiliations:** a Research and Development Group, Hitachi, Ltd, Hitachi Research Laboratory Hitachi Ibaraki 3191292 Japan taigoh.onodera.hg@hitachi.com

## Abstract

Reverse water–gas shift (RWGS) operation at lower temperatures has multiple advantages such as use of low-cost materials and improvement of thermal efficiency. This report demonstrates the enhancement of CO selectivity by Au addition and clarifies the relationship between the enhanced CO selectivity and the density of state (DOS) in the vicinity of the Fermi level (Ef).

## Abstract 2

Reverse water–gas shift (RWGS) operation at lower temperatures has multiple advantages such as use of low-cost materials and improvement of thermal efficiency.

## Graphical abstract

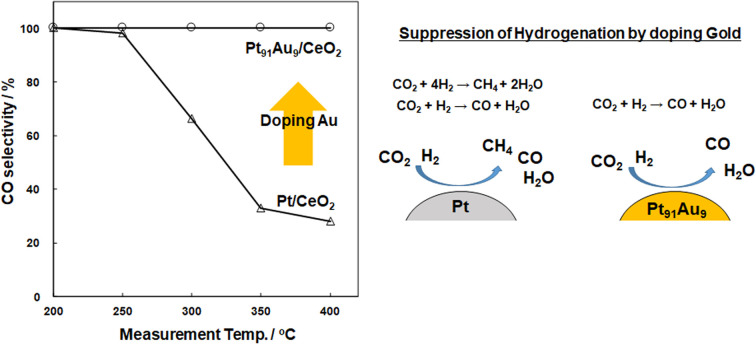

CO_2_ has been recognized as an abundant and inexpensive carbon resource for chemical industry. Reverse water–gas shift (RWGS) reaction is one of the most important reactions in C1 chemistry, because the resulting carbon monoxide (CO) can be utilized as feedstock for the production of valuable compounds such as methanol, dimethyl ether and hydrocarbons.^1–6^

Commonly, RWGS reaction is performed at high temperature to acquire a working performance of CO_2_ conversion and selectivity because of its thermodynamic nature,^7^ as shown in eqn (1).

1 CO_2_ + H_2_ ↔ CO + H_2_O Δ*H* = +41 kJ mol^−1^

On the other hand, RWGS operation at lower temperatures have multiple advantages such as use of low-cost materials and utilization of low-temperature exhaust heat. However, there is the thermodynamic challenge to achieve sufficient level of CO production due to the endothermic nature (eqn (1)). In addition, the Sabatier reaction in eqn (2) proceeds in the same temperature range as RWGS, causing a decrease in CO generation.

2 CO_2_ + 4H_2_ → CH_4_ + 2H_2_O Δ*H* = −165 kJ mol^−1^

Many studies has been reported the improvement of RWGS catalytic activities and CO selectivity such as modifying catalyst metals,^8,9^ catalyst support.^10–12^ Some researchers have reported that Pt catalyst exhibits superior CO_2_ conversion relative to Cu, Fe, and Ni catalysts,^13,14^ but lower CO selectivity.^14^ Therefore, improving CO selectivity of Pt catalyst, it is a fascinating topic, both academically and industrially. It has well-known that Bi-metallic catalysts exhibited higher catalyst activity and selectivity relative to their based metals due to the interaction between the added and based metals.^15,16^ For example, it has been reported that the addition of Ni and CO, which have lower D-band levels than platinum, improves the catalytic activity such as CO selectivity and hydrogenation.^17,18^ On the other hand, adding Au, which has a higher D-band level than Pt, to Pt instead of metals such as Ni and Co, which have a lower D-band level than Pt, and investigating the relationship between the activity and the electronic state is an interesting topic for control of the Pt catalytic activity. Numerous studies have been reported on CO oxidation and CO_2_ reduction reactions in Pt/CeO_2_ and Au/CeO_2_, respectively.^19–23^ However, there is no report on the addition of Au to Pt and the relationship between the electronic state of its valence band and RWGS activity. The purpose of this research is to investigate their relationships and to obtain method for the improvement of CO selectivity.

The Au-doped Pt/CeO_2_ catalysts were synthesized by electroless plating technique with a chelate agent in a manner similar to the previous study.^24^ The starting materials, hexachloroplatinic acid(iv) hexahydrate (0–0.001 mol), hydrogen tetrachloroaurate(iii) tetrahydrate (0.0002–0.001 mol) as metal precursors, cerium oxide (CeO_2_: 140 m^2^ g^−1^, 3.6 g) as a support material, formaldehyde (0.066 mol) as a reducing agent, and citric acid (0.002 mol) as a chelate agent were mixed and stirred with 200 ml of deionized water in the flask. The mixed solution was further stirred for 20 min., and the pH was adjusted to 4 with addition of 1.0 mol l^−1^ NaOH aqueous solution because of lowing pH due to the reduction of Pt and Au ion. After that, the mixed solution was heated to 80 °C and kept the pH at 4 for 3 h. Finally, the mixed solution with the Au-doped Pt/CeO_2_ catalyst (total metal loading: 5 wt%) was filtered with deionized water, and the obtained catalyst was dried at 100 °C in air for 12 h. As a reference, Pt/CeO_2_ catalyst was synthesized by impregnation method.

Fig. 1 shows the XRD patterns of the obtained Au-doped Pt/CeO_2_ catalysts, the change of Pt lattice constant with addition of Au calculated by Vegard's law, and the physical properties of the catalysts. All catalysts presented diffraction peaks at 2*θ* = 28.5°, 33.1°, 47.5°, 56.3°, 59.1°, 69.4°, 76.7°,78.0°, attributed to the fluorite-type CeO_2_ cubic crystal structure.^25^ In addition, the Au-doped Pt/CeO_2_ catalysts except for the Pt/CeO_2_ catalyst shows Au peak at 38.3° and the Pt peak at 39.7°, corresponding to the Au(111) (ref. 26) and Pt(111) (ref. 25) planes. The Pt (111) peak of Pt/CeO_2_ was very broad, suggesting that Pt exists in an amorphous state.

**Fig. 1:**
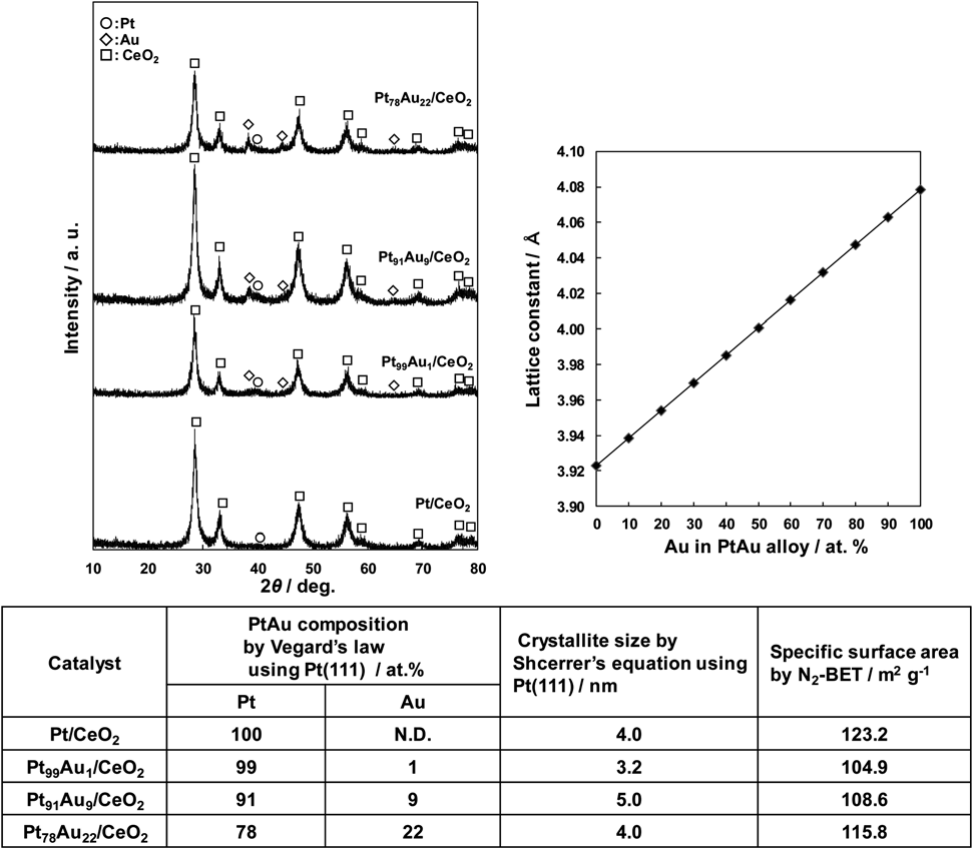
Characterization of the synthesized Pt/CeO_2_ and Au-doped Pt/CeO_2_ catalysts.

The mixing degree of Au in Pt was calculated from Vegard's law using the Pt (111) peak, and it was found that Au was mixed in Pt at a ratio of 1 to 22 at%. The crystallite size of each catalyst calculated from the Pt (111) peak ranged from 3.2 to 5.0 nm, and the specific surface area ranged from 105 to 123 m^2^ g ^−1^, indicating that there were no significant differences among the catalysts.

The catalytic properties of the catalysts for RWGS reaction are shown in Fig. 2. In all catalysts, CO_2_ conversion increased with rising the measurement temperature, being comparable over a temperature range of 200 to 300 °C (Fig. 2a). The CO_2_ conversion had a tendency to increase with decreasing Au content in the catalysts above 350 °C, and the Pt/CeO_2_ catalysts showed the highest CO_2_ conversion activity for all catalysts above 350 °C, exhibiting 45.4% at 400 °C. On the contrary, the CO selectivity of the Pt/CeO_2_ catalyst drastically decreased with increasing the measurement temperature, being 27.9% at 400 °C (Fig. 2b). The CO selectivity showed a tendency to increase with adding Au to Pt, and those at 400 °C of Pt_99_Au_1_/CeO_2_, Pt_91_Au_9_/CeO_2_, Pt_78_Au_22_/CeO_2_ were 40.2%, 100%, 72.0%, respectively. The Pt_91_Au_9_/CeO_2_ catalyst, exhibited the highest CO selectivity, caused little methanation reaction at the range of 200 to 400 °C. These results demonstrate that the Au doping to Pt crystallite structure induces the CO selectivity enhancement of the Pt/CeO_2_ catalyst.

**Fig. 2:**
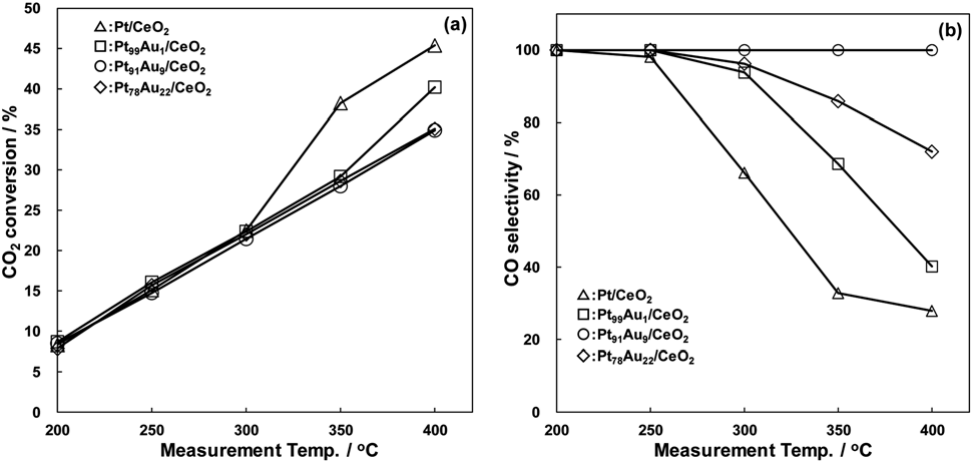
RWGS catalytic properties of the Pt/CeO_2_ and Au-doped Pt/CeO_2_ catalysts, (a) CO_2_ conversion, (b) CO selectivity.

In order to clear the mechanism of the CO selectivity enhancement by addition of Au, the difference of the electronic state in the catalysts were evaluated with measuring the work function. Fig. 3 shows the work function measurement results of the catalysts. In this paper, the work function was evaluated by using photoemission yield spectroscopy in air (PYSA) instrument (AC-3, RIKEN KEIKI Co., Ltd) with an open counter as an electron detector and a D2 lamp as an ultraviolet light source, being calculated by using the leading edge of an electron emission current from the catalysts surface. The work functions of the Pt/CeO_2_, Pt_99_Au_1_/CeO_2_, Pt_91_Au_9_/CeO_2_, Pt_78_Au_22_/CeO_2_ were 6.2, 5.2, 5.2, and 5.2 eV, respectively. The Pt/CeO_2_ catalyst exhibited bigger work function compared to the others, and the work function was also bigger than the literature value of Pt (5.65 eV).^27^ This result indicates that Pt surface in the Pt/CeO_2_ was oxidation state. On the other hand, the work functions of the Au-doped Pt catalysts were same values. Comparing the electron emission amount of the Au-doped Pt catalyst at 7.0 eV, the order was Pt_99_Au_1_/CeO_2_ > Pt_78_Au_22_/CeO_2_ > Pt_91_Au_9_/CeO_2_, and the higher the CO selectivity, the lower the electron emission. This result suggests that the difference in CO selectivity may be due to the difference in the electron-donating properties of the catalysts. So, to clarify the relationship between the CO selectivity and the electron emission amount from the catalysts, the core level and valence bands of each catalyst were analyzed by using X-ray photoelectron spectroscopy (XPS, PHI QuanteraSXM) with Al-Kα (1486.6 eV) monochromator.

**Fig. 3:**
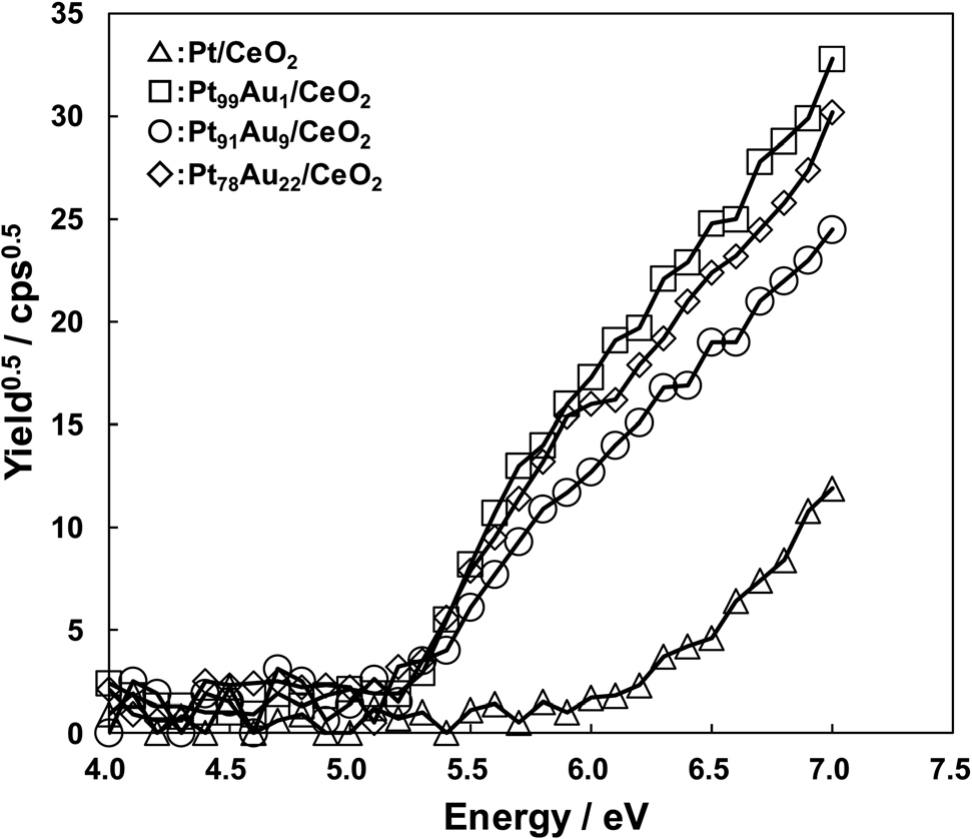
Work function of the Pt/CeO_2_ and the Au-doped Pt/CeO_2_ catalysts.

The measurement results of Pt 4f core level XPS spectra of each catalyst are shown in Fig. 4. XPS peaks at binding energy values of 71.3 eV (Pt 4f_7/2_) and 74.7 eV (Pt 4f_5/2_) for the Pt_91_Au_9_/CeO_2_ and Pt_78_Au_22_/CeO_2_ catalysts were attributed to metallic Pt species on the surface. XPS peaks at slightly higher binding energy values than that of metallic Pt were observed for the Pt_99_Au_1_/CeO_2_ catalyst. On the other hand, XPS peaks at binding energy values of 73.0 eV (Pt 4f_7/2_) and 75.8 eV (Pt 4f_5/2_) for the Pt/CeO_2_ shifted to higher binding energy values than that of metallic Pt and were attributed to Pt oxide species on the surface. This XRS results are consistent with the measurement results of work function for each catalyst.

**Fig. 4:**
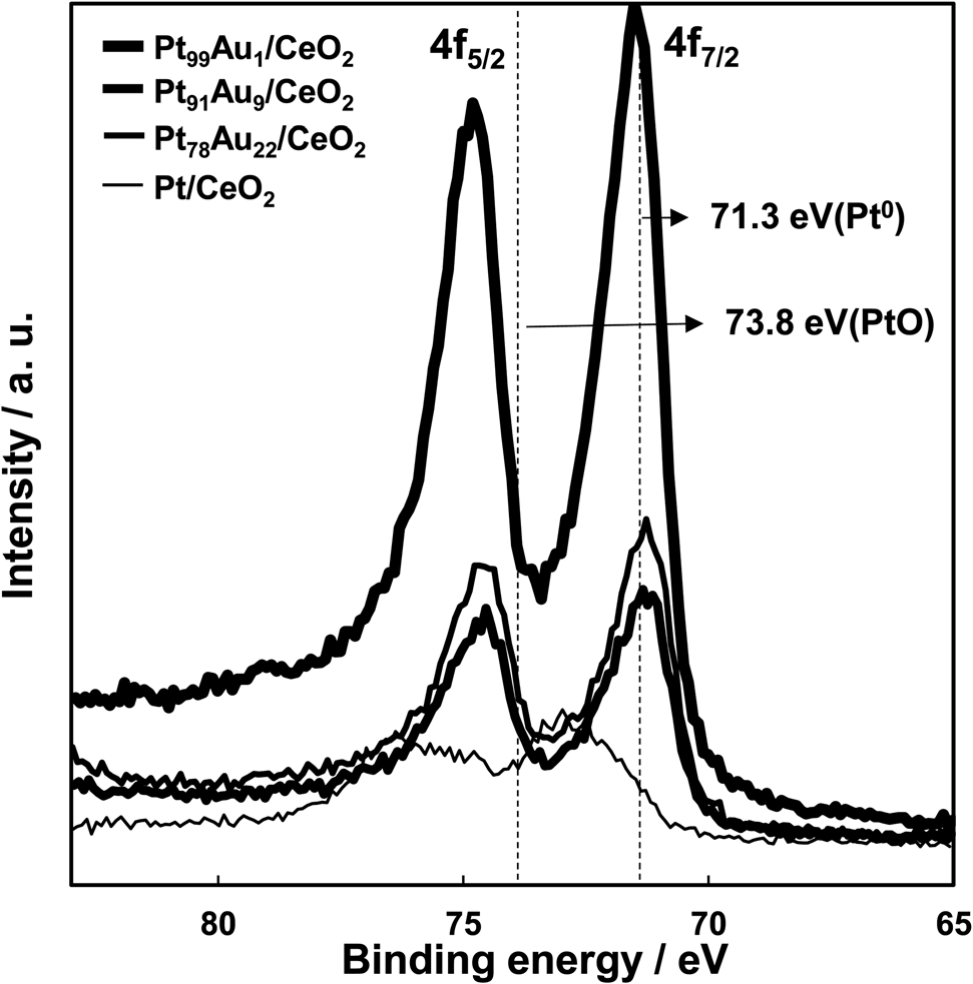
Pt 4f core level spectra of the Pt/CeO_2_ and the Au-doped Pt/CeO_2_ catalysts.

Fig. 5 shows the valence band spectra of the catalysts. The valence band spectra of all catalysts were observed the O 2p band corresponding to the CeO_2_ support spread over 3.0–9.0 eV.^28^ The Pt and Au have high density of state (DOS) near the *E*_f_ (0 eV), and the valence band spread at the range of 0–3.0 eV. The DOS at the range of 0–3.0 eV of the Pt/CeO_2_ catalyst was much lower than those of the others because the Pt existed oxidation state. On the other hand, the catalysts except for the Pt/CeO_2_ catalyst have high DOS at the range of 0–3.0 eV, and the DOS order was Pt_99_Au_1_/CeO_2_ > Pt_78_Au_22_/CeO_2_ > Pt_91_Au_9_/CeO_2_. On the contrary, the CO selectivity order at 400 °C was Pt_91_Au_9_/CeO_2_ > Pt_78_Au_22_/CeO_2_ > Pt_99_Au_1_/CeO_2_, and the lower DOS at the range of 0–3.0 eV led to the higher CO selectivity in the Au-doped Pt/CeO_2_ catalysts. When the Au-doped Pt/CeO_2_ catalyst has higher DOS at the range of 0–3.0 eV, more electrons go to an antibonding orbital of CO_2_. Therefore, the O–C–O bonds are weakened which tend to give rise to methanation reaction. It is suggested that a low DOS near the Fermi level may enhance the CO selectivity of the RWGS reaction.

**Fig. 5:**
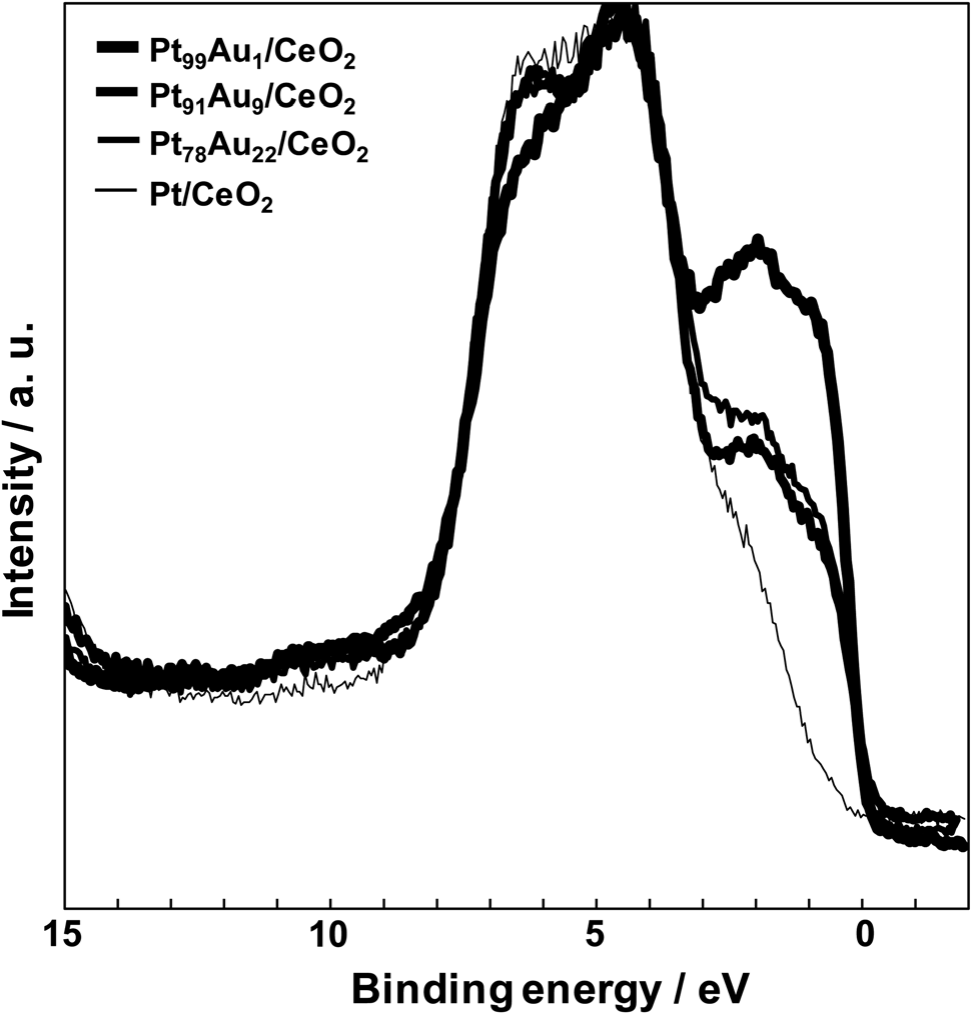
Valence band spectra of the Pt/CeO_2_ and the Au-doped Pt/CeO_2_ catalysts.

In conclusion, relationship between the RWGS catalytic properties and electronic state of the Au-doped Pt/CeO_2_ catalysts were investigated. The Au-doped Pt/CeO_2_ catalysts, synthesized by electroless plating technique, exhibited the comparable CO_2_ conversion activities to the Pt/CeO_2_ catalyst up to 300 °C. In addition, the CO selectivity of the Au-doped Pt/CeO_2_ catalysts were much higher than that of the Pt/CeO_2_ catalyst above 250 °C. The work function and the valence band analysis indicated that the DOS in the vicinity of the fermi level of the Au-doped catalysts was associated with the CO selectivity of the Au-doped Pt/CeO_2_ catalyst. The addition ot the appropriate Au to the Pt/CeO_2_ catalyst formed the advantageous DOS for RWGS reaction. On the other hand, when the DOS is higher, the O–C–O bonds are weakened due to the increase in electron donation from the Au-doped Pt/CeO_2_ catalyst to an antibonding orbital of CO_2_ and promotes the methanation reaction. It is suggested that the higher CO selectivity of the Pt/CeO_2_ catalyst may realizes by controlling the DOS in the vicinity of the Fermi level.

## Conflicts of interest

There are no conflicts to declare.

## Supplementary Material
